# Editorial: Computational methods for multi-omics data analysis in cancer precision medicine

**DOI:** 10.3389/fgene.2023.1226975

**Published:** 2023-07-05

**Authors:** Moein Piroozkhah, Radman Mazloomnejad, Zahra Salehi, Ehsan Nazemalhosseini-Mojarad

**Affiliations:** ^1^ Basic and Molecular Epidemiology of Gastrointestinal Disorders Research Center, Research Institute for Gastroenterology and Liver Diseases, Shahid Beheshti University of Medical Sciences, Tehran, Iran; ^2^ Department of Immunology, School of Medicine, Tehran University of Medical Sciences, Tehran, Iran; ^3^ Gastroenterology and Liver Diseases Research Center, Research Institute for Gastroenterology and Liver Diseases, Shahid Beheshti University of Medical Sciences, Tehran, Iran

**Keywords:** computational methods, multi-omics data analysis, precision medicine, cancer, machine learning

## Introduction

Multi-omics constitutes a broad realm of biomedical research that covers the different levels of organisms, from genomics to higher levels, such as proteomics or metabolomics, and the interactions among these levels (Boroń et al., 2022). A commonality among all these levels is epistemological complexity. This indicates that investigations in this field necessitate a reduction in complexity to provide a better elaboration of a biological system. Thanks to ever-increasing computational methods to handle multi-omics data. Computational methods have facilitated many new avenues of research, from basic biological topics to possible applications in drug development or molecular systems engineering in heterogeneous diseases such as cancers, and opened the way for approaching the ideal of personalized medicine in the clinical oncology (Ayton et al., 2022). The articles of this Research Topic provide an up-to-date summary of novel computational methods of multi-omics data analysis to address the state-of-the-art and future perspectives in the field of precision oncology (Supplementary Table S1). The authors utilized a variety of data sources and their combinations, demonstrating the importance of multimodal data analysis in the research of diverse cancers.

## “Pan-cancer” analysis

Lately, there has been a lot of attention on pan-cancer analyses, and several online databases and analytical tools have gained popularity. Some of these tools include GEPIA2, UALCAN, cBioPortal, TIMER2.0, TISIDB, STRING, DAVID, HPA, and GeneMANIA (Xie et al., 2022). These resources have played a crucial role in advancing our understanding of pan-cancer research by enabling researchers to access and analyze comprehensive datasets, perform sophisticated analyses, and uncover valuable insights. In the context of prognostic implications for cancer patients, Eid et al. employed online web tools to investigate the protein WDR12, which demonstrates heterogeneous expression patterns across diverse cancer types. Significant associations between WDR12 expression profiles and various cancer-related factors highlight its potential as a robust prognostic biomarker and prospective target for innovative cancer treatments. In a similar vein, Liu et al. conducted a pan-cancer investigation of NFE2L3, a transcription factor known for its regulatory role in the expression of genes associated with cellular homeostasis and response to oxidative stress, utilizing diverse online databases. The study revealed consistent overexpression of NFE2L3 across a wide range of human tumors. The implications of NFE2L3 in critical cellular processes and its correlations with various molecular characteristics underscore its potential as a promising molecular biomarker for the diagnosis and prognosis of pan-cancer, as well as a prospective target for tumor treatment. Moreover, ANLN, a conserved cytoskeletal protein, plays a critical role in regulating cytokinesis and contractile ring formation during cell division. Through the utilization of online databases, Liu et al. present evidence indicating the increased expression of ANLN across diverse tumor types, its association with tumor cell proliferation, migration, infiltration, and prognosis, as well as its impact on tumor immune evasion.

To die or not to die that is not a question for eukaryotic cells. The issues that really matter are WHEN and HOW to die. Cell death has been observed for quite a long time; nevertheless, nowadays, different kinds of cell death are identified, such as apoptosis, pyroptosis, necroptosis, and cuproptosis. Cell death is associated with the development of many diseases, with tumors as a notable example (Tong et al., 2022). Zheng et al. employed analytical packages to study the Gasdermin-D (GSDM) family, proteins implicated in pyroptosis, and discovered significant upregulation of GSDM genes across various cancer types. Utilizing the Wilcoxon rank-sum test, they analyzed expression differences between malignant and corresponding normal samples for each cancer type. Moreover, the authors conducted an in-depth assessment of the pan-cancer tumor microenvironment (TME) using the CIBERSORT algorithm. The findings revealed strong correlations between GSDM gene expression levels and prognosis, clinical characteristics, TME features, and stemness scores, especially in urinary system cancers, indicating their possible role in carcinogenesis. This study conducted a drug sensitivity analysis, which revealed potential therapeutic implications that could be derived from the findings. In a distinct pan-cancer analysis, Jiang et al. investigated STC2, a secreted glycoprotein related to calcium, glucose homeostasis, and phosphorus metastasis. Their study using the “Limma” package disclosed elevated STC2 expression and association. Furthermore, survival analysis employing “survminer” and “survival” packages demonstrated adverse outcomes and significant correlations with tumor immune microenvironment, immune cell infiltration, immune checkpoint genes, mismatch repair genes, tumor mutation burden, microsatellite instability, and drug sensitivity.

## “Gastrointestinal cancer” analysis

Researchers have been trying to push back the frontiers of cancer pathophysiology knowledge in recent years by investigating the roles of N7-methylguanosine (m^7^G), a novel post-transcriptional modifier of RNAs (Luo et al., 2022). In this Research Topic, Wei et al. focused on hepatocellular carcinoma (HCC) and utilized LASSO regression to develop and strengthen an immune prognostic signature on the basis of m^7^G-related lncRNA data. Eventually, the nine m^7^G-related-lncRNA risk model was verified *in silico* through training and testing sets (*n* = 219 and 146 LIHC patients, respectively), and its prognostic accuracy was measured using ROC curves, univariate and multivariate Cox regression analysis. In a comparable manner, Chen et al. established an extensive risk model for colorectal cancer (CRC) through the application of multivariate Cox and LASSO Cox regression analysis. The analysis successfully identified an eleven m^7^G-related DNA damage repair signature that predicts patient prognosis. Moreover, the “CIBERSORT” and “MCPcounter” packages were also used to analyze the different immune microenvironment statuses between high- and low-risk groups. The constructed risk model was useful not only for assessing patients’ prognosis but even providing further insights into immunotherapy strategies in CRC. By using similar methodologies, Hong et al. constructed and assessed a prognostic signature consisting of seven m^7^G-associated miRNAs that could accurately predict patient outcomes and direct personalized therapy.

In gastric cancer (GC), Yuan et al. established the apoptosisScore to quantify the apoptosis index of each patient by using “principal component analysis.” Besides the prognostic potential, this score is also correlated with immune infiltrates and sensitivity to immunotherapy. This study flow encourages the mining of prognostic usage of cell death-related gene signatures in other types of cancers. Regarding HCC, Peng et al. implemented the Wilcoxon test to detect necroptosis-related lncRNAs with differential expression from the TCGA database. Based on the analysis result, they screened prognostic lncRNAs and established ten necroptosis-related lncRNA signatures. The risk score was then calculated utilizing Lasso–Cox stepwise regression analysis. This signature is involved in immune cell infiltration and the expression of immune checkpoints and can be utilized to predict immunotherapeutic efficacy. These results were also consolidated by *in vivo* data. Using similar approaches, the prognostic potential of cuproptosis was extended to KIRC by Hong et al., who proposed a prognosis signature made up of four cuproptosis-related LncRNAs.

The identification of potential targets or signatures, such as the proposed lncRNA signatures, may offer valuable insights. However, it is important to note that these findings are not sufficient by themselves to establish clinically applicable tools. Several challenges exist when it comes to understanding the role of lncRNA in cancers, including the need to define which lncRNAs and modules within them interact with effector proteins and convey target specificity (Mattick et al., 2023). LncRNAs can play complex roles associated with diverse hallmarks of cancer. As such, a more comprehensive understanding of cell and developmental biology, as well as gene-environment interactions, can be gleaned by studying the roles of lncRNAs and how they function in dynamic assemblies with other macromolecules (Winkle et al., 2021). Additionally, larger and more rigorous clinical studies are necessary to validate the diagnostic and prognostic value of lncRNAs and to assess their potential as therapeutic targets.

As far as cholangiocarcinoma is concerned, many efforts should be made to improve the prognosis of this poor-outcome cancer. One of the promising attempts was performed by Wang et al. They constructed empirical Bayes and Markov random field models eLBP to determine cell communication-related genes using single-cell RNAseq data, with value for prognostic prediction and chemotherapeutic decision-making in cholangiocarcinoma patients.

One important consideration in cancer research may be the influence of sex on the incidence and survival rates of cancers. Pursuing the answer to this question: “Sex, possible culprits or accomplices for pancreatic cancer (PC)?”, Ramezankhani et al. leveraged multiple microarray expression data for PC to determine the down and upregulated genes between the sex that were also specified for androgen and estrogen receptors. By the use of several online databases and analytic tools, including DAVID, Enrichr, bioDBnet, pancreatic expression database, TRANSFAC, HIPPIE, and BisoGenet, they identified the importance of the androgenic effectors in tumorigenesis, such as the potential role of testosterone in the extracellular matrix–cell interaction. To determine the relationship between abnormal purine/uric acid metabolism with the prognosis of HCC patients, Yang et al. used gene set variation analysis and stratified 371 HCC patients (The transcriptome sequencing data and corresponding clinical information of HCC patients were downloaded from TCGA database) into four groups: high purine biosynthesis and high purine metabolism (PBhiPMhi), high purine biosynthesis and low purine metabolism, low purine biosynthesis, and high purine metabolism, and low purine biosynthesis and low purine metabolism (PBloPMlo). The survival study indicated that the PBhiPMhi group had the poorest prognosis, whereas the PBloPMlo group had the best. In Zou et al.’s and Zhao et al.’s research articles, respectively, the readers can find explicit information on a “seven-cancer driver gene signature” was constructed for the projection of survival and tumor immunity in HCC; and how by assessing the glycosylation patterns will be helpful in identifying the characteristics of immune cell infiltration and selecting accurate treatment methods in HCC patients.

Perhaps one of the best ways to construct a prognostic risk score model is employing the weighted gene co-expression network analysis (WGCNA) to create co-expression networks and identify co-expression modules associated with clinical features. In the next step, these modules are utilized for further analysis. An interesting example of this approach was done by Wang et al. in GC. First, 200 overlapping coagulation-related genes (CRGs) were discovered by GEO and AmiGO2 (a gene ontology database). Then, they applied WGCNA and identified two module genes containing 141 CRGs. Finally, a Coagulation-score risk model was constructed using LASSO regression, which was an independent predictor of overall survival for GC patients. Last but not least, the potency of the *EIF2S2* gene in HCC as a prognostic factor, which is closely related to immune infiltration and immune checkpoints in HCC patients, was declared in the Liu et al. study, by the use of TCGA transcriptomic data. They also employed Genomics of Drug Sensitivity in Cancer database and figured out the *EIF2S2* high expression group was more sensitive to Paclitaxel and Sunitinib.

## “Breast and gynecological cancer” analysis

Breast cancer (BC) is a heterogeneous disease influenced by RNA modification-associated proteins (RMPs) (Chang et al., 2020). In recent studies, Wang et al. and Li et al. employed computational techniques, including univariate Cox regression, differential expression analysis, and LASSO regression, to explore the prognostic value and interrelationships of RMPs in BC. Wang et al. identified four prognosis-related genes (PRGs) with the highest prognostic value, and their prognostic models incorporating these PRGs revealed alterations in biological pathways, genomic mutations, immune infiltrations, RNAss scores, drug sensitivities, and prognostic implications. This comprehensive analysis shed light on the collective functions and features of diverse RMPs in BC, enabling accurate prediction of clinical outcomes using advanced forecasting models. On the other hand, Li et al. established a lactate-related long non-coding RNA prognostic signature (LRLPS) using Cox and LASSO regression methods. The LRLPS showed consistent and autonomous prognostic capability and revealed differences in immune-related pathways, immune infiltration, and responsiveness to immunotherapeutic interventions between high- and low-risk BC patient cohorts. These findings contribute to our understanding of the intricate connections between BC and RMPs, providing valuable insights for personalized prognostic patterns.

In another study, Li et al. utilized consensus clustering analysis, facilitated by the “ConsensusCluster Plus” R package, to identify distinct cuproptosis-clusters. They subsequently developed a novel prognostic model using univariate Cox regression analysis, multivariate Cox regression analysis, and the stepwise Akaike information criterion (stepAIC) to select relevant variables for the model construction. By examining 13 CRRs, they revealed expression differences between BC and normal tissues. Incorporating five prognostic CRRs, BC patients were classified into two distinct cuproptosis-clusters (C1 and C2), with C2 exhibiting superior survival outcomes and increased immune infiltration.

In addition, gynecologic cancers also present significant clinical challenges that necessitate innovative strategies for understanding their underlying mechanisms and enhancing patient outcomes. Sharbatoghli et al. applied non-invasive prenatal testing (NIPT) to investigate copy number variations (CNVs) in circulating tumor DNA (ctDNA) from ovarian cancer patients undergoing neoadjuvant chemotherapy (NAC). Six OC patients were categorized into NAC-sensitive and NAC-resistant groups, and CNV analysis was performed using two NIPT methods. WISECONDORX revealed fewer CNV changes in NAC-sensitive patients, while NextGENe identified CNVs in both coding and non-coding genes exclusively in NAC-resistant patients. Analysis of these genes demonstrated their amplification and a significant association between their high expression levels and reduced overall survival in chemotherapy-resistant patients.

Using the “ConsensClusterPlus” R package for consensus clustering, Chen et al. classified Uterine Corpus Endometrial Carcinoma samples based on CRG expression. Principal component analysis estimated molecular pattern distribution, while gene set variation analysis assessed biological process changes. Differentially expressed genes were identified, and a CRG score system was developed. Immunophenoscores evaluated the correlation between CRG scores and immunotherapy efficacy. Lower CRG scores indicated improved prognosis, enhanced immunotherapeutic response, and increased tumor mutation burden. Moreover, in cervical cancer, a cuproptosis-related lncRNA signature has been developed, demonstrating strong potential for survival prediction, immunotherapy assessment, and prognosis post-radiotherapy. Liu et al. constructed this signature through a LASSO-Cox analysis of lncRNAs associated with cuproptosis, which has proven robust in stratifying patients based on risk scores, contributing to improved prognostication and potential biomarker identification.

## “Skin cutaneous melanoma” analysis

Recent research has identified the complement protein C1Q as a crucial factor in the pathogenesis and progression of cutaneous melanoma. Yang et al. conducted a study demonstrating that the increased expression of C1QA, C1QB, and C1QC subunits of C1Q holds substantial diagnostic and prognostic value in cutaneous melanoma. Elevated expression of these subunits was associated with improved patient survival and served as independent biomarkers. Furthermore, increased expression levels correlated with immune cell infiltration, expression of other biomarkers, immune checkpoint proteins, and enrichment of immune and apoptotic pathways. These findings suggest that upregulated C1QA, C1QB, and C1QC may serve as potential biomarkers for the diagnosis and prognosis of cutaneous melanoma, particularly concerning the response to immunotherapy.

## “Glioma” analysis

Glioma, a formidable type of brain cancer, poses substantial challenges in diagnosis and treatment. Xuan et al. conducted a groundbreaking study elucidating the crucial role of silent information regulator (SIRT) family enzymes in vital cellular processes, including apoptosis, metabolism, aging, and cell cycle regulation, with implications for glioma and other cancers. Through innovative analyses employing LASSO regression and multivariate Cox methods, they identified a novel SIRT-based gene signature that accurately stratifies glioma patients based on transcriptome and clinical data. Prognostic evaluations confirmed its exceptional predictive value and broad applicability. An overall survival nomogram, incorporating sex, age, risk score, pathological grade, and IDH status, was developed to aid clinical decision-making. Notable distinctions in immune status and immune cell infiltration between high and low-risk groups shed light on the relationship between the SIRT signature and the tumor microenvironment. This SIRT-based signature offers a potent tool for glioma prognosis and personalized management, potentially improving patient outcomes.

## Conclusion

In conclusion, this Research Topic emphasizes the paramount importance of computational methods in revolutionizing cancer precision medicine through the analysis of multi-omics data. Although numerous studies have employed single-mode data, it is imperative to emphasize the increasing importance of the multi-omics concept in cancer research. In this context, the integration of diverse data types, including genomics, epigenomics, transcriptomics, and proteomics, is vital to gaining a more comprehensive understanding of cancer biology and enabling the identification of novel biomarkers, prognostic indicators, and therapeutic targets.

The key findings discussed herein underscore the efficacy of diverse computational techniques, including network analysis and pathway-based approaches. These methodologies have demonstrated their potential in predicting patient outcomes, facilitating treatment decisions, and optimizing personalized therapeutic strategies. Despite the progress made in multi-omics research, it is worth noting that many studies have predominantly focused on implementing omics studies alongside each other rather than genuinely integrating multi-modal data. It is essential to address this limitation and further promote the development and application of advanced computational methods that can effectively integrate and analyze multi-modal data. By doing so, the cancer research community can unlock the full potential of multi-omics approaches, leading to breakthroughs in precision medicine and improved patient care.

## Prospects

The forthcoming years will prioritize delving into single-cell multi-omics analysis, incorporating real-time data for dynamic treatment monitoring, and integrating electronic health records into multi-omics frameworks. As a subfield of artificial intelligence (AI), machine-learning methods have an exceptional capability to integrate Omics data effectively. Machine-learning algorithms are trained to model complex patterns that cannot be accurately captured by traditional mathematical models in high-dimensional data. Even though there has been a notable surge in the number of multi-modal experiments conducted and an abundance of data at our disposal for analysis, the potential of machine learning tools in effectively integrating these datasets has not been fully harnessed. It is imperative that future research delves into the vast capacities of utilizing machine learning, particularly deep learning, in conducting multi-modal data analysis. Eventually, establishing standardized data formats and user-friendly software tools, as well as promoting collaboration between computational and clinical researchers, are essential steps toward the practical application of molecular cancer discoveries in clinical settings.
