# Supplementary material for: Editorial: Computational methods for multi-omics data analysis in cancer precision medicine
Source: Front Genet. 2023 Jul 5;14:1226975. doi: 10.3389/fgene.2023.1226975 (PMC10354637; doi:10.3389/fgene.2023.1226975)
Supplement: Supplementary file 1 [file Table1.docx]

**Table S1 | Summaries of Computational methods for multi-omics data analysis in cancer precision Medicine**

| **No.** | **Title** | **Cancer** | **Aim** | **Method(s)** | | **Omics** | **Result(s)** | **Authors** |
| --- | --- | --- | --- | --- | --- | --- | --- | --- |
|  |  |  |  | **Data / Databases** | **Statistical analysis: Algorithms, R packages, etc.** |  |  |  |
| 1 | **Integrative analysis of WDR12 as a potential prognostic and immunological biomarker in multiple human tumors** | Pan-cancer | To investigate the potential oncogenic effects of WDR12 in multiple human cancers | • TCGA database  • GEO database  •TIMER2 database  • GEPIA2 database  • UALCAN database  • HPA database  • cBioPortal database  • SMART database  • MEXPRESS database  • TCGA database database  • TISIDB database  • STRING database  • DAVID database | NA | Genomics  Epigenomics  Transcriptomics  Proteomics  Immunomics | • WDR12 could be a potential prognostic biomarker for pan-cancer. | [Eid R et al.](https://www.frontiersin.org/articles/10.3389/fgene.2022.1008502/full) |
| 2 | **Multi-Omics Analysis of Molecular Characteristics and Carcinogenic Effect of NFE2L3 in Pan-Cancer** | Pan-cancer | a comprehensive pan-cancer analysis to systematically investigate the role of NFE2L3 in tumorigenesis and cancer progression. | • HPA database  • Cancer Cell Line Encyclopedia database  • TIMER2.0 database  • TCGA database  • SMART database  • UCSC Xena database  • The Protter database  • cBioPortal database  • TIMER database  • TISIDB database  • TIDE website  • CancerSEA database  • GeneMANIA database  • STRING database  • GEPIA2 database | NA | Epigenomics  Transcriptomics  Proteomics  Immunomics | • NFE2L3 could be a potential prognostic biomarker for pan-cancer. | [Liu M et al.](https://www.frontiersin.org/articles/10.3389/fgene.2022.916973/full) |
| 3 | **Pan-cancer analysis of the prognostic and immunological role of ANLN: An onco-immunological biomarker** | Pan-cancer | to investigate the relationship between ANLN and various aspects of tumor biology, including immune infiltration, immune evasion, tumor progression, immunotherapy, and tumor prognosis. | • TCGA database  •UALCAN database  • HPA database  • PROTTER database  • STRING database  • GEPIA database  • MethSurv database  • cBioPortal database  • TIMER2.0 database  • TIDE website | NA | Genomics  Epigenomics  Transcriptomics  Proteomics  Immunomics | • Increased ANLN expression in multiple tumors is associated with tumor cell proliferation, migration, infiltration, and prognosis. ANLN methylation and genetic alteration are also associated with prognosis in numerous tumors. ANLN facilitates tumor immune evasion through different mechanisms, and may serve as a predictive biomarker for the response to immune checkpoint inhibitors. | [Liu K et al.](https://www.frontiersin.org/articles/10.3389/fgene.2022.922472/full) |
| 4 | **A systematic pan-cancer analysis of the gasdermin (GSDM) family of genes and their correlation with prognosis, the tumor microenvironment, and drug sensitivity** | Pan-cancer | to undertake a comprehensive evaluation of the GSDM family's involvement in the context of pan-cancer. | • TCGA database  • GEO database  • UCSC Xena project, •cBioPortal database  •Kaplan–Meier Plotter  • CellMiner database | • Wilcoxon rank-sum test  • CIBERSORT algorithm  • Spearman's method  • R packages:  ‘limma’ | Epigenomics  Transcriptomics  Immunomics | • GSDM genes were significantly upregulated in various cancer types and associated with clinical characteristics and chemotherapy drug sensitivity, indicating their potential as therapeutic targets for pan-cancer treatment. | [Zheng Y et al.](https://www.frontiersin.org/articles/10.3389/fgene.2022.926796/full) |
| 5 | **A Pan-Cancer Analysis Reveals the Prognostic and Immunotherapeutic Value of Stanniocalcin-2 (STC2)** | Pan-cancer | to investigate the expression profile, prognostic value, and potential immunotherapeutic implications of STC2 in cancer patients. | • TCGA database  • GEO database  • UCSC XENA database (TCGA, GTEx)  • HPA database  • CellMiner database  • COMPARTMANTS  • cBioPortal database | • R packages:  ‘limma’  ‘survminer  ‘survival’ | Genomics  Transcriptomics  Immunomics | • The pan-cancer analysis presented herein demonstrates that STC2 holds significant prognostic value across various cancer types and may serve as a potential immunotherapeutic target. | [Jiang Z-H et al.](https://www.frontiersin.org/articles/10.3389/fgene.2022.927046/full) |
| 6 | **Prognostic Signature and Tumor Immune Landscape of N7-Methylguanosine-Related lncRNAs in Hepatocellular Carcinoma** | LIHC | To identify the relationship between m7G-related lncRNAs with the prognosis of LIHC patients | • TCGA database  • GDSC database | • Pearson correlation  • Univariate regression  • multivariate Cox regression  • LASSO regression  • KM analysis  • ROC analysis  • R packages:  “DESeq2”  “glmnet.”  “clusterProfiler.”  “GSVA”  “maftools”  “survival”  “survminer”  “pRRophetic,” | Genomics  Transcriptomics | The m7G-related lncRNA risk model might display the potential value in predicting prognosis, immunotherapy response, and drug sensitivity in LIHC patients. | [Wei Wei et al.](https://doi.org/10.3389/fgene.2022.906496) |
| 7 | **Construction and validation of a necroptosis-related lncRNAs prognosis signature of hepatocellular carcinoma** | LIHC | To investigates the link among necroptosis-related lncRNA, prognosis, immune microenvironment, and immunotherapy response in LIHC patients | • TCGA database  • Ensemble database | • Pearson correlation  • Wilcoxon method  • Univariate Cox regression  • LASSO Cox stepwise regression  • Functional enrichment analysis  • CIBERSORT algorithm  • ssGSEA  • R packages:  “tidyverse”  “dplyr”  “ggbiplot”  “bioconductor limma”  “ggalluvial”  “ClusterProfiler”  “ggplot2”  “enrichplot”  “GOplot”  “ConsensusClusterPlus”  “vioplot” | Transcriptomics | The 10 necroptosis-related lncRNAs signature, is valuable for survival prediction and holds promise as prognostic markers for LIHC. | [YunZhen Peng et al.](https://doi.org/10.3389/fgene.2022.916024) |
| 8 | **m^7^G-Related DNA Damage Repair Genes are Potential Biomarkers for Predicting Prognosis and Immunotherapy Effectiveness in Colon Cancer Patients** | CRC | To explore whether m7G-related DNA damage repair genes may be used as biomarkers to predict the prognosis of colon cancer patients. | • GSEA database  • TCGA database  • GEO database  • David database  • TIDE database | • NMF  • DCA curves  • PCA  • Nomogram  • KM analysis  • ROC analysis  • Multivariate Cox  • LASSO Cox regression  • ssGSEA  • R packages:  ‘psych’  ‘NMF’  “limma”  “GSVA”  “pheatmap”  “survival”  ‘rms’  “CIBERSORT”  “MCPcounter”  “ggplot2”  “tidyverse”  “maftools”  “pRRophetic” | Transcriptomics | The m^7^G-related DDR genes can be used as important markers for predicting patient prognosis and immunotherapy response. | [Shuran Chen et al.](https://doi.org/10.3389/fgene.2022.918159) |
| 9 | **Apoptosis-Related Gene-Mediated Cell Death Pattern Induces Immunosuppression and Immunotherapy Resistance in Gastric Cancer** | GC | To clarify relationships between apoptosis-related genes with immunosuppression, and immunotherapy resistance of GC patients. | • TCGA database  • GEO database  • cBioPortal database | • Multivariate Cox regression  • PCA  • KM analysis  • ROC analysis  • Functional enrichment analysis  • ESTIMATE algorithm  • R packages:  “Bioconductor“  “limma”  “reshape2”  “ConsensusClusterPlus”  “Survival”  “estimated”  “cluster Profiler”  “Rich plot”  “ggplot2” | Transcriptomics | Establishing apoptosisScore as a prognostic biomarker, correlated with immune infiltrates, and sensitivity to immunotherapy in GC. | [Xiaolu Yuan et al.](https://doi.org/10.3389/fgene.2022.921163) |
| 10 | **Comprehensive analysis of the cancer driver genes constructs a seven-gene signature for prediction of survival and tumor immunity in hepatocellular carcinoma** | LIHC | To constructed a prognosis score based on CDGs, which could predict the survival of HCC patients | • TCGA database  • ICGC dataset | • Wilcoxon test  • GSEA  • LASSO regression  • Stepwise multivariate COX regression  • KM analysis  • ROC analysis  • R packages:  “Maftools”  “clusterProfiler”  “Survival”  “SurvivalROC”  “GSVA” | Genomics  Transcriptomics | This study underlined the importance of CDGs in LIHC and provided a strategy for patient stratification for precise medication. | [Jun Zou et al.](https://doi.org/10.3389/fgene.2022.937948) |
| 11 | **A Novel Purine and Uric Metabolism Signature Predicting the Prognosis of Hepatocellular Carcinoma** | LIHC | To identify relationship between abnormal purine and uric acid metabolism with prognosis of LIHC patients. | • TCGA database  • GEO database  • STRING database | • Univariable Cox regression  • KM analysis  • ROC analysis  • Functional enrichment analysis  • R packages:  “GSVA”  “limma”  “Survival” | Transcriptomics | The level of peripheral blood uric acid in patients with HCC is correlated with their prognosis | [Shengjie Yang et al.](https://doi.org/10.3389/fgene.2022.942267) |
| 12 | **Construction and validation of a novel coagulation-related 7-gene prognostic signature for gastric cancer** | GC | To construct a coagulation-related gene signature and prognostic model for GC. | • TCGA database  • GEO database  • UCSC Xena database  • AmiGO 2 database  • TIMER database | • WGCNA  • LASSO Cox regression  • KM analysis  • ROC analysis  • Nomogram  • ssGSEA  • R packages:  “WGCNA”  “glmnet”  “stats”  “Survival”  “KMsurv”  “survivalROC”  “RMS”  “GSVA”  “ggpubr” | Transcriptomics | Coagulation-related gene models provide new insights and targets for the diagnosis, prognosis prediction, and treatment management of GC patients. | [Bofang Wang et al.](https://doi.org/10.3389/fgene.2022.957655) |
| 13 | **Gender-related differentially expressed genes in pancreatic cancer: possible culprits or accomplices?** | PC | Identification of gene candidates as the possible link between gender and PC progression or survival rates may help in developing strategies to reduce the incidence of this cancer. | • GEO database  • TCGA database  • DAVID database  • bioDBnet database  • PED database  • TRANSFAC database  • STRING database  • HIPPIE dataset  • BisoGenet | • Functional enrichment analysis  • GEO2R | Transcriptomics | The plausible importance of the androgenic effectors in tumorigenesis, such as the androgen-regulated expression of the GLI transcription factor and the potential role of testosterone in the extracellular matrix (ECM)–cell interaction, which are known for their importance in tumorigenesis. | [Roya Ramezankhani et al.](https://doi.org/10.3389/fgene.2022.966941) |
| 14 | **Integrating cell interaction with transcription factors to obtain a robust gene panel for prognostic prediction and therapies in cholangiocarcinoma** | CCA | To explore critical cell signaling and biomarkers induced *via* cell communication during immune exhaustion in CCA. | • GEO database  • ENCODE database  • ChEA3 database  • TRANSFAC database  • CellChat database | • Empirical Bayes  • Markov random field models  • eLBP  • KM analysis  • Functional enrichment analysis  • R packages:  “ConsensusClusterPlus”  “GSVA”  “limma”  “estimate”  “minfi”  “conumee”  “survival”  “glmnet” | Transcriptomics | The TMRS panel is a reliable tool for prognostic prediction and chemotherapeutic decision-making in CCA. | [Tingjie Wang et al.](https://doi.org/10.3389/fgene.2022.981145) |
| 15 | **EIF2S2 is a novel independent prognostic biomarker and correlated with immune infiltrates in hepatocellular carcinoma** | LIHC | To identify the role of EIF2S2 in the malignant progression of liver cancer and its relationship with immune infiltration. | • TCGA database  • TIMER2. 0 database  • HPA database  • GDSC database | • Functional enrichment analysis  • Univariate COX regression  • Multivariate COX regression  • ROC analysis  • R packages:  “survminer”  “timeROC”  “ClusterProfilter”  “pRRophetic” | Transcriptomics | The EIF2S2 gene can be used as a prognostic factor for LIHC, which is closely related to immune infiltration and immune checkpoints, and may play a potential regulatory role in predicting drug sensitivity. | [Jing Liu et al.](https://doi.org/10.3389/fgene.2022.992343) |
| 16 | **Characterization of glycosylation regulator-mediated glycosylation modification patterns and tumor microenvironment infiltration in hepatocellular carcinoma** | LIHC | To investigate the roles of glycosylation in LIHC tumorigenesis and progression | • TCGA database  • GEO database  • GDSC database  • xCell  • MSigDB database | • ssGSEA  • Univariate Cox regression  • Consensus clustering algorithm  • PCA  • R packages:  “limma”  “surviviner”  “survival”  “ConsensusClusterPlus”  “nomogramEx”  “clusterProfiler”  “IOBR”  “pheatmap”  “GSVA”  “pRRophetic” | Transcriptomics | Evaluating the glycosylation patterns of patients with HCC will be helpful in identifying the characteristics of immune cell infiltration and selecting accurate treatment methods. | [Linlin Zhao et al.](https://doi.org/10.3389/fgene.2022.1001901) |
| 17 | **A Novel M7G-Related MicroRNAs Risk Signature Predicts the Prognosis and Tumor Microenvironment of Kidney Renal Clear Cell Carcinoma** | KIRC | To establish a risk signature on the foundation of m7G-associated miRNAs, which can precisely forecast the prognosis of KIRC patients. | • TCGA database  • UCSC Xean browser  • TargetScan  • TIDE website  • STRING database | • Univariable Cox regression  • Lasso Cox regression  • Multivariable Cox regression  • KM analysis  • ROC analysis  • CIBERSORT  • ESTIMATE  • ssGSEA algorithms  • R packages:  “edgeR”  “rms”  “pRRophetic” | Genomics  Transcriptomics | Based on m7G-related miRNAs, a risk signature was successfully constructed, which could precisely forecast the prognosis of sufferers and guide personalized immunotherapy for KIRC patients. | [Peng Hong et al.](https://doi.org/10.3389/fgene.2022.922358) |
| 18 | **Prognostic value and immunological characteristics of a novel cuproptosis-related long noncoding RNAs risk signature in kidney renal clear cell carcinoma** | KIRC | To build a prognosis signature based on CRLs, which can achieve accurate prediction of the outcome of KIRC patients. | • TCGA database  • UCSC Xean browser  • TIDE website | • Univariate Cox regression  • Lasso Cox regression  • Multivariate Cox regression  • KM analysis  • ROC analysis  • GSEA  • CIBERSORT-ABS, CIBERSORT, EPIC, MCPCOUNTER, QUANTISEQ, TIMER, XCELL  ESTIMATE  • R packages:  “limma”  “ggpubr”  “maltools”  “glment”  “survival”  “survminer”  “rms”  “clusterProfiler”  “enrichplot”  “pRRophetic” |  | The four identified risk LncRNAs (especially APCDD1L-DT and MINCR) can be novel targets for immunotherapy of KIRC patients | [Peng Hong et al.](https://doi.org/10.3389/fgene.2022.1009555) |
| 19 | **Cross-talk of four types of RNA modification proteins with adenosine reveals the landscape of multivariate prognostic patterns in breast cancer** | Breast cancer (BC) | to investigate the interplay and potential synergies among RNA-modified proteins (RMPs) in generating multivariate prognostic signatures for breast cancer (BC) and develop diverse RMP-associated prognostic models to predict BC clinical outcomes. | • TCGA database  • GEO database  • METABRIC database | • Univariate Cox regression  • Differential expression  • LASSO regression  • Multivariate Cox regression. | Genomics  Transcriptomics  Immunomics | • Diverse prognostic models were developed to predict breast cancer clinical outcomes using RNA-modified proteins (RMPs). | [Wang X et al.](https://www.frontiersin.org/articles/10.3389/fgene.2022.943378/full) |
| 20 | **A lactate-related LncRNA model for predicting prognosis, immune landscape and therapeutic response in breast cancer** | Breast cancer (BC) | To construct a lactate-related lncRNAs prognostic signature (LRLPS) for BC patients to predict prognosis, tumor microenvironment, and treatment responses. | • TCGA database  • GEO database | • Univariate Cox regression  • LASSO regression  • Multivariate Cox regression | Genomics  Transcriptomics  Immunomics | • A prognostic prediction model for breast cancer patients, called the LRLPS, was developed based on 7 LRLs and demonstrated excellent and robust prognostic prediction ability. | [Li J et al.](https://www.frontiersin.org/articles/10.3389/fgene.2022.956246/full) |
| 21 | **The cuproptosis-related signature predicts prognosis and indicates immune microenvironment in breast cancer** | Breast cancer (BC) | to examine the association between cuproptosis and breast cancer (BC) and develop a prognostic signature based on cuproptosis-related factors to effectively predict BC prognosis. | • UCSC XENA  • METABRIC database  • cBioportal  • MSigDB  • TCGA database  • GEO database | • Univariate Cox regression  • Multivariate Cox regression  • Stepwise Akaike information criterion (stepAIC)  R packages:  "ConsensusCluster Plus" | Genomics  Transcriptomics  Immunomics | • A cuproptosis-related prognostic signature and nomogram were developed, which could predict BC prognosis, tumor immune microenvironment, and response to immunotherapy. | [Li J et al.](https://www.frontiersin.org/articles/10.3389/fgene.2022.977322/full) |
| 22 | **Copy Number Variation of Circulating Tumor DNA (ctDNA) Detected Using NIPT in Neoadjuvant Chemotherapy-Treated Ovarian Cancer Patients** | Ovarian cancer | to explore copy number variations (CNVs) using non-invasive prenatal testing in plasma circulating tumor DNA (ctDNA) from ovarian cancer (OC) patients who received neoadjuvant chemotherapy (NAC) treatment. | • TCGA database  • GEO database | • non-invasive prenatal testing (NIPT)  • WISECONDORX  • NextGENe | Genomics  Transcriptomics | • The results suggest that CNVs detected through non-invasive prenatal testing (NIPT) in circulating tumor DNA (ctDNA) may serve as potential markers of clinical response to neoadjuvant chemotherapy (NAC) treatment. | [Sharbatoghli et al.](https://www.frontiersin.org/articles/10.3389/fgene.2022.938985/full) |
| 23 | **Cuproptosis patterns and tumor microenvironment in endometrial cancer** | Uterine Corpus Endometrial Carcinoma (UCEC). | To investigate the relationship between cuproptosis-related genes (CRGs), tumor microenvironment (TME), and the prognosis of UCEC patients. | • TCGA databsse  • GEO database  • TCIA database | • Single-sample gene-set enrichment analysis (ssGSEA)  • R packages:  “limma”  “ggplot2”  "ConsensClusterPlus" | Genomics  Transcriptomics  Immunomics | • The study identified three clusters of cuproptosis-related genes (CRGs) and three gene clusters based on differentially expressed genes (DEGs) with significant prognostic differences in UCEC patients. | [Chen J et al.](https://www.frontiersin.org/articles/10.3389/fgene.2022.1001374/full) |
| 24 | **Construction and comprehensive analysis of a curoptosis-related lncRNA signature for predicting prognosis and immune response in cervical cancer** | Cervical cancer | To create a cuproptosis-related lncRNA signature for predicting survival, immunotherapy, and prognosis in patients with cervical cancer | • TCGA database | • The Perl programming language  • Pearson correlation analysis  • ‘limma,’ ‘ggplot2’, ‘ggalluvial’, ‘dplyr’, and ‘clusterProfiler’ R packages  • Univariate Cox regression  • Multivariate Cox regression  • LASSO regression  • Nomogram and principal component analysis  •TIMER, CIBERSORT, CIBERSORT-ABS, QUANTISEQ, MCPCOUNTER, and XCELL algorithms  • R packages:  ‘limma,’  ‘ggplot2’  ‘ggalluvial’  ‘dplyr’  ‘clusterProfiler’  ‘GSVA’  ‘GSEABase’  ‘survival’  ‘survivor’  ‘survival’  ‘pheatmap’  ‘ggpubr’ | Genomics  Transcriptomics  Immunomics | • A cuproptosis-related long non-coding RNA (lncRNA) signature was developed and found to have favorable predictive potential for cervical cancer patients. | [Liu L et al.](https://www.frontiersin.org/articles/10.3389/fgene.2023.1023613/full) |
| 25 | **Prognostic and immune-related value of complement C1Q (C1QA, C1QB, and C1QC) in skin cutaneous melanoma** | Skin cutaneous melanoma (SKCM) | to investigate the impact of C1QA, C1QB, and C1QC expression on tumor immunity and prognosis in cutaneous melanoma. | • GEPIA  • TCGA database  • GEO database  • TIMER database  • HPAdatabase  • LinkedOmics database | NA | Genomics  Transcriptomics  Immunomics | • Overexpression of C1QA, C1QB, and C1QC has significant diagnostic value in cutaneous melanoma (SKCM) and is associated with better overall survival (OS). | [Yang H et al.](https://www.frontiersin.org/articles/10.3389/fgene.2022.940306/full) |
| 26 | **Constructing a signature based on the SIRT family to help the prognosis and treatment sensitivity in glioma patients** | Glioma | To identify a new gene signature based on SIRT family genes for risk assessment and stratification of glioma patients | • TCGA database  • CGGA database  • UCSC Xena database  • HPAdatabase  • cBioPortal database | • LASSO regression  • Kaplan-Meier analysis  • ROC curve analysis  • Univariate Cox regression  • Multivariate Cox regression  • ssGSEA analysis | Genomics  Transcriptomics  proteomics  Immunomics | • A gene signature consisting of five SIRT family genes was developed and found to accurately predict overall survival (OS) in glioma patients. | [Xuan F et al.](https://www.frontiersin.org/articles/10.3389/fgene.2022.1035368/full) |

BC: Breast cancer; CCA: cholangiocarcinoma; CDGs: Cancer driver genes; CRC: Colorectal Cancer; CRLs: cuproptosis-related LncRNAs; DDR: DNA damage repair; eLBP: empirical Bayes loopy belief propagation; GC: Gastric Cancer; GDSC: Genomics of Drug Sensitivity in Cancer; GEO: Gene Expression Omnibus; HPA: Human Protein Atlas; ICGC: International Cancer Genome Consortium; KIRC: kidney renal clear cell carcinoma; KM: Kaplan–Meier; LASSO: Last absolute shrinkage and selection operator; LIHC: Liver Hepatocellular Carcinoma; NMF: non-negative matrix factorization; PC: Pancreatic cancer; PCA: Principal component analysis; PED: Pancreatic Expression Database; ROC: Receiver operating characteristic; ssGSEA: Single-sample gene-set enrichment analysis; TCGA: The Cancer Genome Atlas; TMRS: Tumor microenvironment risk score; WGCNA: Weighted gene co-expression network analysis
